# The temporal and spatial endophytic fungal community of *Huperzia serrata*: diversity and relevance to huperzine A production by the host

**DOI:** 10.1186/s12866-022-02702-y

**Published:** 2022-11-24

**Authors:** Zhuhui Shen, Xubing Liu, Jia Yang, Yanli Wang, Kai Yao, Qingmiao Huo, Yanping Fu, Yahui Wei, Bin Guo

**Affiliations:** Shaanxi Provincial Key Laboratory of Biotechnology; Key Laboratory of Resource Biology and Biotechnology in Western China, Ministry of Education, Northwest University, Xi’an, 710069 Shaanxi China

**Keywords:** Endophytic fungi, Temporal and spatial distribution, Huperzine A, *Huperzia serrata*

## Abstract

**Background:**

Plants maintain the steady-state balance of the mutually beneficial symbiosis relationship with their endophytic fungi through secondary metabolites. Meanwhile endophytic fungi can serve as biological inducers to promote the biosynthesis and accumulation of valuable secondary metabolites in host plants through a variety of ways. The composition and structure of endophytic fungal community are affected by many factors, including tissues, seasons and so on. In this work, we studied the community diversity, temporal and spatial pattern of endophytic fungi detected from the roots, stems and leaves of *Huperzia serrata* in different seasons. The correlation between endophytic fungi and huperzine A (HupA) content in plants was analyzed.

**Results:**

A total of 7005 operational taxonomic units were detected, and all strains were identified as 14 phyla, 54 classes, 140 orders, 351 families and 742 genera. Alpha diversity analysis showed that the diversity of endophytic fungi in stem and leaf was higher than that in root, and the diversity in summer (August) was lower than that in other months. NMDS analysis showed that the endophytic fungal communities of leaves, stems and roots were significantly different, and the root and leaf communities were also different between four seasons. Through correlation analysis, it was found that 33 genera of the endophytic fungi of *H. serrata* showed a significant positive correlation with the content of HupA (*p* < 0.05), of which 13 genera (*Strelitziana, Devriesia, Articulospora, Derxomyces, Cyphellophora, Trechispora, Kurtzmanomyces, Capnobotryella, Erythrobasidium, Camptophora, Stagonospora, Lachnum, Golubevia*) showed a highly significant positive correlation with the content of HupA (*p* < 0.01). These endophytic fungi may have the potential to promote the biosynthesis and accumulation of HupA in plant.

**Conclusions:**

This report is the first time to analyze the diversity of endophytic fungi in tissues of *H. serrata* in different seasons, which proves that there is variability in different tissues and seasonal distribution patterns. These findings provide references to the study of endophytic fungi of *H. serrata.*

**Supplementary Information:**

The online version contains supplementary material available at 10.1186/s12866-022-02702-y.

## Introduction

*Huperzia serrata* (Thunb. ex Murray) Trev., also known as Qian Ceng Ta, is a perennial medicinal fern in Lycopodiaceae. It has the curative effects of hemostasis, removing blood stasis, detoxification and treatment of schizophrenia [1–4]. Alkaloids, triterpenoids and flavonoids are the main effective ingredients of *H. serrata* [5].

Huperzine A (HupA), isolated from *H. serrata* in 1986, has been proved to be a highly selective and reversible acetylcholinesterase inhibitor with a new chemical structure, and has a strong efficacy in the treatment of Alzheimer disease (AD) and myasthenia gravis [4, 6, 7]. HupA has been approved as a drug for the treatment of AD in China [8] and is used as a supplement to prevent further memory degradation in the United States [9]. However, the content of Hup A in wild *H. serrata* plants is low (0.0046%-0.0133%) [10], and the plants grow slowly, which is difficult to meet the market demand. Overexploitation and habitat fragmentation have made *H. serrata* an endangered plant in China [11, 12]. At present, the chemical synthesis of HupA is not suitable for industrial production [13], as is the artificial cultivation technology and tissue culture technology of *H. serrata* [14].

Endophytic fungi reside in cells or intercellular spaces at a certain life stage or throughout the life cycle in healthy plants, inducing hidden and asymptomatic infections in plant tissues, without causing disease symptoms. Among them, fungi that have a dormant or latent period in plant tissues before causing disease symptoms on the host plants are still endophytic fungi, although these fungi are clearly pathogenic; pathogenic fungi that are parasitic in plants but where disease symptoms do not appear after infection are also endophytic fungi [15]. Endophytic fungi obtain most of the nutrients from the host plants and provide ecological benefits to the plants in return. Apparent host benefits include improved tolerance to heavy metals, increased drought resistance, reduced herbivory, systemic resistance against pathogens, and generally enhanced growth [16]. In addition, endophytic fungi also promote the production of secondary metabolites of host plants by stimulating key genes in the plant biosynthesis pathway and synthesizing enzymes that can convert precursors to active compounds or their analogues [17–19]. It is reported that the content of HupA produced by plants varies from different tissues and seasons [10]. At present, researchers have isolated some endophytic fungi, which produce HupA, from *H. serrata* and other *Phlegmariurus* by using conventional methods of isolation and purification of endophytic fungi [20–34]. Several studies have also shown that endophytic fungal community composition is correlated with plant tissues and seasons [35–37]. Therefore, it is necessary to study the diversity and composition of endophytic fungi of *H. serrata*.

In this study, we explored the temporal and spatial diversity of endophytic fungal community of *H. serrata*. Furthermore, endophytic fungi with significant positive correlation between HupA content of *H. serrata* were screened. It provides more reference to a clearer understanding of the fungal community ecology of this important medicinal plant.

## Results

### Classification and distribution of endophytic fungi

**Fig. 1:**
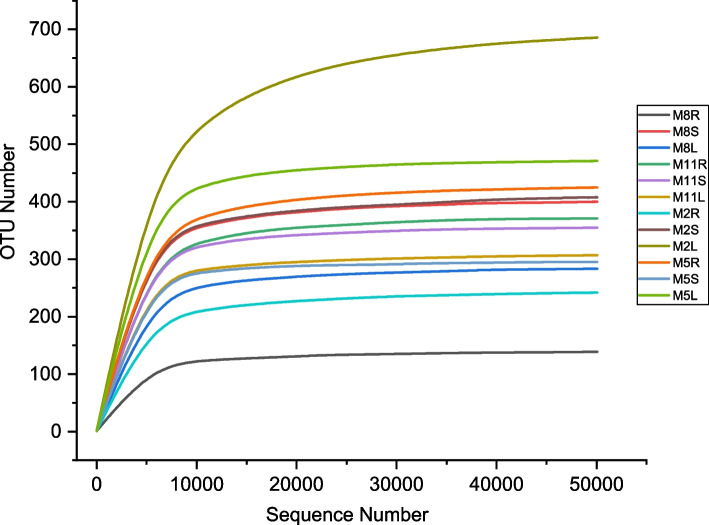
Rarefaction curves of OTUs in 12 groups of samples (M8, August; M11, November; M2, February; M5, May; R, root; S, stem; L, leaf)

**Fig. 2:**
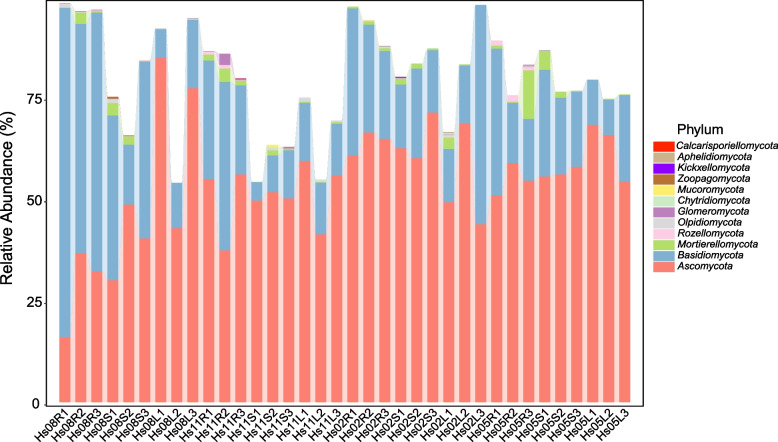
Relative abundance of fungal phylum in 36 samples of *H. serrata* (excluding unclassified and unidentified phyla)

A total of 7005 operational taxonomic units (OTUs) were detected in 36 samples(4 seasons × 3 tissues × 3 replicates)of *H. serrata*. In all samples, the rarefaction curves tended to be flat, indicating that the sequencing depth was sufficient (Fig. 1). According to ITS sequence classification, all strains were identified as in 14 phyla, 54 classes, 140 orders, 351 families, 742 genera (including unclassified and unidentified groups). At the phylum level, Ascomycota was the dominant phylum, followed by Basidiomycota (Fig. 2), and their relative abundance was 54% and 25% respectively (Fig. S1). The main classes of Ascomycota were Eurotiomycetes (20%), Dothideomycetes (12%) and Leotiomycetes (11%). The main classes of Basidiomycota were Agaricomycetes (11%) and Tremellomycetes (8%) (Fig. S1).

**Fig. 3:**
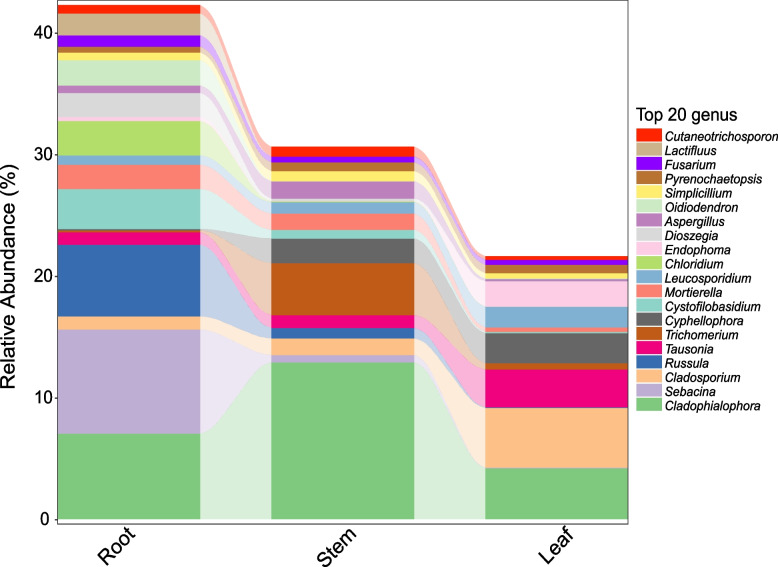
Relative abundance of fungal genera in root, stem and leaf samples of *H. serrata* (top 20 genera of the total)

At the genus level, *Cladophialophora* (8%), *Sebacina* (3%), *Cladosporium* (2%), *Russula* (2%), *Tausonia* (2%), *Trichomerium* (2%) and *Cypellophora* (2%) were the top 7 genera with high abundance, and they were distributed among different tissues in different seasons (Fig. S1). The dominant endophytic fungal groups (genera with relative abundance > 2%) varied with tissues and seasons. In terms of tissue distribution, the dominant genera of the leaf, stem and root were all in the top 20 of the total relative abundance, of which *Cladophialophora* was the common dominant genus in the three tissues. The dominant genera in the leaf were *Cladosporium, Cladophialophora, Tausonia, Cypellophora* and *Endophora*. The dominant genera in the stem were *Cladophialophora, Trichomerium* and *Cypellophora*. The dominant genera in the root were *Sebacina, Cladophialophora, Russula, Cystofilobasidium, Chloridium* and *Oidiodendron*. *Latifluus* was a fungal genus restricted to roots (Fig. 3).

**Fig. 4:**
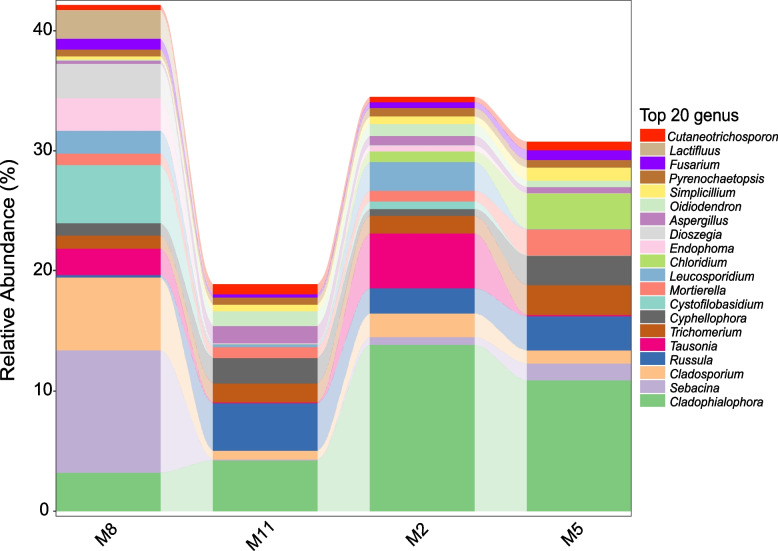
Relative abundance of fungal genera in August (M8), November (M11), February (M2) and May (M5) samples (top 20 genera of the total)

In terms of seasonal distribution, the dominant genera of February, May, August and November were all in the top 20 of the total relative abundance, of which *Cladophialophora* was the common dominant genus in four months. The dominant fungal genera in February were *Cladophialophora, Tausonia, Leucosporidium,* and *Russula*. In May, the dominant fungal genera were *Cladophialophora, Chloridium, Russula, Trichomerium, Cyphellophora* and *Mortierella*. In August, the dominant fungal genera were *Sebacina, Cladosporium, Cystofilobasidium, Cladophialophora, Dioszegia, Endophoma, Lactifluus* and *Tausonia.* In November, the dominant fungal genera were *Cladophialophora, Russula* and *Cyphellophora. Latifluus* was a fungal genus found only in the August sampling. *Chloridium* was not detected in the samples of August (Fig. 4).

### Diversity of endophytic fungi

**Fig. 5:**
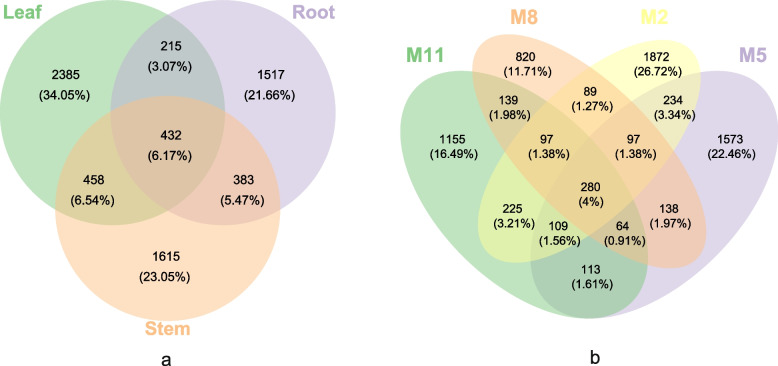
Venn diagrams showing number of shared OTUs among sample groups. **a** Number of shared OTUs among the root, stem and leaf samples associated with *H. serrata*. **b** Number of shared OTUs among the August (M8), November (M11), February (M2) and May (M5) samples

**Fig. 6:**
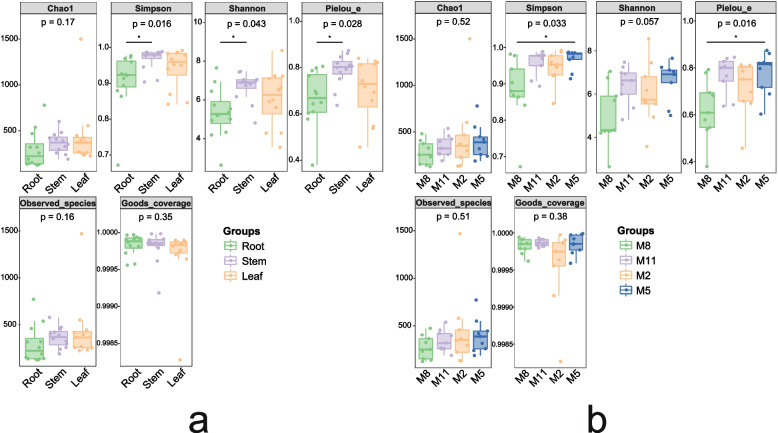
**a** Alpha diversity indices of endophytic fungi in different tissue samples. **b** Alpha diversity indices of endophytic fungi in different season samples (M8, August; M11, November; M2, February; M5, May). The number under the diversity index label is the *p* value of Kruskal Wallis test. The asterisk on the line in the figure indicates that there is a significant difference between the two groups (*p* < 0.05)

A total of 7005 OTUs were detected in all ITS libraries, of which less than 10% were found in common for all three tissues (Fig. 5a). The numbers of unique and common OUTs for the three tissues were leaf > stem > root. The endophytic fungal communities in three tissues were calculated for alpha diversity indices. The depth index (Goods coverage) of each sample library was more than 99.9%, indicating that the sampling was reasonable. Shannon, Simpson's and Pielou's evenness indexes of stems were significantly higher than those of roots (Fig. 6a). In general, all diversity indexes showed that the highest richness, diversity and evenness of endophytic fungal community was from the stem, followed by the leaf, and the lowest was the root.

Of the detected OTUs, less than 5% were found in common for all four months (Fig. 5b). Overall, the total and unique OTUs in the four months were M2 > M5 > M11 > M8. Alpha diversity values of the endophytic fungal communities in the four months were calculated. Simpson's index and Pielou's evenness index in M5 were significantly higher than it in M8 (Fig. 6b). This indicates that the richness, diversity and evenness of endophytic fungi were highest in M5 and lowest in M8.

### The temporal and spatial patterns of endophytic fungal community

**Fig. 7:**
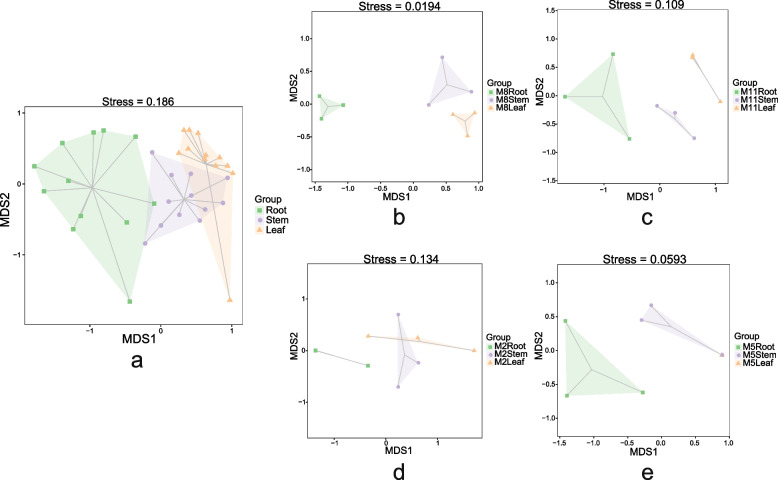
NMDS analysis of fungal communities among three tissues samples of *H. serrata* in different seasons (**a**, four seasons; **b**, M8, August; **c**, M11, November; **d**, M2, February; **e**, M5, May)

The similarity between endophytic fungi in different tissues and seasons was analyzed by Non-metric multidimensional scaling (NMDS). The results showed that there were highly significant differences (*p* = 0.001) in endophytic fungi between different tissues (Fig. 7a). The results of four months' analysis showed that there were highly significant differences (*p* < 0.01) in the community distribution of endophytic fungi between the three tissues in August and November (Fig. 7b, c). In February and May, the endophytic fungal communities of leaf and stem were similar, and they were significantly different from those of root (*p* < 0.05) (Fig. 7d, e).

**Fig. 8:**
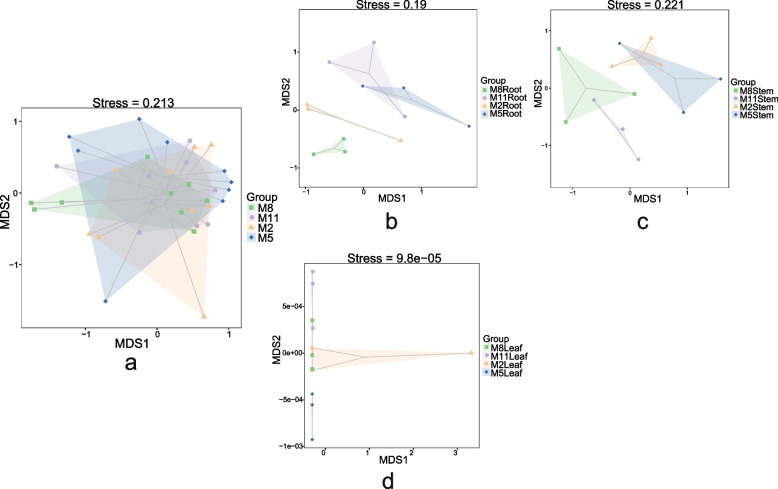
NMDS analysis of fungal communities among four seasons (M8, August; M11, November; M2, February; M5, May) samples of *H. serrata* in different tissues (**a**, three tissues; **b**, root; **c**, stem; **d**, leaf)

Adonis results showed that there were significant differences (*p* = 0.024) in fungal communities of endophytic fungi between different months, but NMDS analysis showed that stress more than 0.2 (Fig. 8a). Therefore, we analyzed the three tissues independently, and the endophytic fungal communities of the three tissues were significantly different (*p* < 0.05) between the four months. The results of NMDS analysis showed that the grouping results of roots and leaves were more reliable (stress < 0.2) (Fig. 8b, d), while the fungal communities of stems were not reliable for grouping between the four months (Fig. 8c). The fungal communities in roots were similar in November and May. The fungal communities of leaves were similar in February, August and November. The fungal communities of stems in August and November were similar, those in February and May were similar.

### Correlation between endophytic fungal communities and HupA content of *H. serrata*

**Fig. 9:**
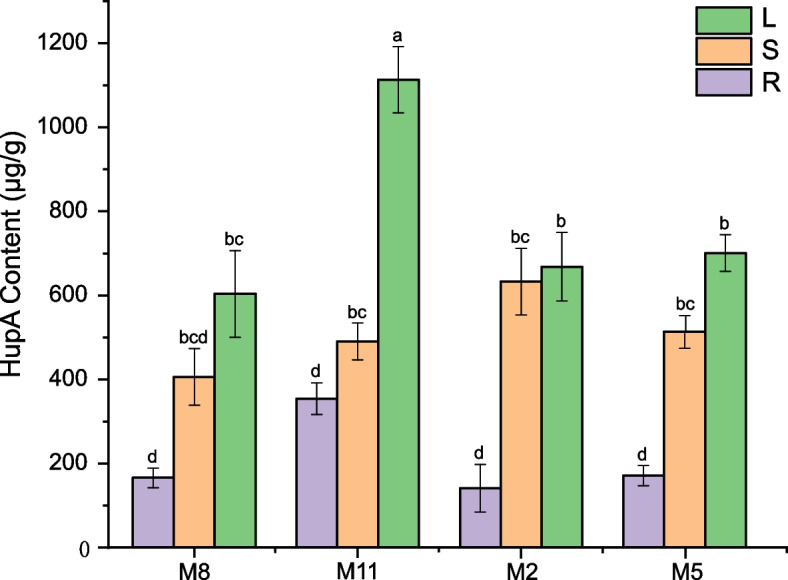
Content of HupA in *H. serrata* (M8, August; M11, November; M2, February; M5, May; R, root; S, stem; L, leaf)*.* Different letters above the bars indicate statistically significant (*p* < 0.05) differences according to Tukey's Method tests. Error bars represent SE

The contents of HupA in 36 samples of *H. serrata* (4 seasons × 3 tissues × 3 replicates) were determined (Fig. 9). Considering same month of sampling, HupA content was highest in leaves, followed by stems and roots. The HupA content of leaves in November was highest.

The correlation between the contents of HupA with endophytic fungal communities was further analyzed and the Spearman correlation index was calculated. It was found that 33 genera of the endophytic fungi of *H. serrata* showed a significant positive correlation with the content of HupA (*p* < 0.05), of which 13 genera showed a highly significant positive correlation with the content of HupA (*p* < 0.01) (Table 1).

**Table 1:** The positive correlation of endophytic fungi with HupA content within *H. serrata* (*p* < 0.05)

Genus	Spearman’s correlation coefficient	Significance(p)
*Strelitziana*	0.776542	0
*Devriesia*	0.644937	0.000022
*Articulospora*	0.643924	0.000023
*Derxomyces*	0.640771	0.000026
*Cyphellophora*	0.579162	0.000215
*Trechispora*	0.556435	0.000425
*Kurtzmanomyces*	0.474750	0.003438
*Capnobotryella*	0.453266	0.005500
*Erythrobasidium*	0.448894	0.006030
*Camptophora*	0.446763	0.006304
*Stagonospora*	0.445764	0.006436
*Lachnum*	0.440435	0.007181
*Golubevia*	0.435129	0.007996
*Phyllosticta*	0.421009	0.010558
*Taphrina*	0.409584	0.013113
*Colacogloea*	0.395349	0.017009
*Bannoa*	0.393780	0.017492
*Lepiota*	0.393608	0.017546
*Arachnopeziza*	0.391924	0.018079
*Helminthosporium*	0.384183	0.020705
*Marasmius*	0.381949	0.021519
*Cryptocoryneum*	0.379425	0.022472
*Pestalotiopsis*	0.375937	0.023845
*Pseudeurotium*	0.371951	0.025498
*Meira*	0.361083	0.030493
*Alternaria*	0.357805	0.032148
*Herpotrichia*	0.349738	0.036536
*Carlosrosaea*	0.349643	0.036590
*Proliferodiscus*	0.347363	0.037917
*Lactarius*	0.341633	0.041423
*Tylospora*	0.339846	0.042568
*Arthrinium*	0.339309	0.042918
*Moesziomyces*	0.339130	0.043034

## Discussion

This is the first study where endophytic fungal communities of *H. serrata* were analyzed during four seasons. A total of 7005 OTUs were identified, belonging to 14 phyla, 54 classes, 140 orders, 351 families and 742 genera. Ascomycota was the dominant phylum, followed by Basidiomycota, and their abundance accounted for 79% of the communities. At the genus level, the dominant genera of endophytic fungi in each sample were different. *Cladophialophora* (8%) was the dominant genus in different seasons and different tissues. *Latifluus* was a unique genus of root samples of August.

The number of endophytic fungi identified from the three tissues was leaf > stem > root. The highest richness and diversity of endophytic fungal community was in stems, followed by leaves and roots. This result is different from previous reports [38–40], which may be due to the fact that endophytic fungi were identified from *H. serrata* in different seasons, and the differences of endophytic communities in tissues are shaped by the factors such as plant species, soil type, geographic, and environmental conditions [41]. NMDS analysis showed that there were significant differences between endophytic fungal communities when all four seasons were considered in root, stem and leaf. However, in February and May, the endophytic fungal communities of leaf were similar to that of stem. This was consistent with the reported results [38–40]. The NMDS analysis of the same tissue across four months revealed that there were significant differences in the communities of root and leaf, but the analysis of stem communities provided a poor representation. Since the fungal diversity of stem was higher than that of leaf and root, the analysis of whole plant communities across four months was largely influenced by the distribution of endophytic fungal communities in the stem. So far, we have come to the conclusion that the distribution of endophytic fungi community of *H. serrata* varies with tissues and seasons.

Alpha diversity analysis showed that the richness and diversity of endophytic fungi was the lowest in summer (August) and the highest in spring (May). The variation in a seasonal pattern of fungal colonization might be notably associated with the seasonal behavior of endophytic fungi. In seasonal studies, some endophytic fungi detected from summer or autumn had high diversity. It was suggested that the high humidity and high temperature in the rainy season are conducive to the growth and diffusion of fungal spores [37, 42]. However, some studies have reported that the diversity of endophytic fungi is higher in winter [43–45]. Since spring, a large number of fungal species in *H. serrata* have gradually been in a dynamic equilibrium through continuous interspecific competition. The dominant position of dominant species was gradually strengthened. The relative abundance of the top 20 genera in August is higher than that of other three months. Therefore, the richness and diversity index in August are low. With the decrease of temperature, the physiological activity of endophytic fungi decreased, interspecific competition was not active in winter, and species diversity began to increase. However, this inference needs further research to verify.

Up to now, about 16 fungal genera have been reported to produce HupA [20–34], of which 13 genera were found in this report. Their relative abundance was the highest in leaves (*Acremonium*, *Alternaria*, *Arthrinium*, *Botrytis*, *Cladosporium*, *Colletotrichum*, *Podospora*), followed by stems (*Aspergillus*, *Leptosphaeria*, *Mucor*, *Penicillium*), and the lowest in roots (*Fusarium*, *Trichoderma*)(Fig. S2a). In the distribution of months, there were 10 genera (*Alternaria*, *Cladosporium*, *Colletotrichum*, *Fusarium*, *Leptosphaeria*, *Mucor*, *Acremonium*, *Arthrinium*, *Aspergillus*, *Penicillium*) with higher relative abundance in August and November (Fig. S2b). The climate of August where the plant materials were collected is suitable for the growth of endophytic fungi producing HupA [46, 47]. *Cladosporium* was dominant in leaf samples of August. So, for future isolations, at least from this field site, the probability of successful isolation in summer is higher.

The accumulation of alkaloids is an important chemical defense strategy for plants to adapt to environmental stress [48–50], and the defense response of plants is usually triggered by the perception of endophytic fungi [51, 52]. The content of HupA in plants was consistent with the trend of the endophytic fungi presence, across tissues and seasons. Thus, the existence of some special fungal species might be one key factor that induced the accumulation of HupA in *H. serrata*. Spearman analysis showed positive correlation of 33 endophytic fungal genera with the content of HupA (*p* < 0.05), with 13 genera as highly significant (*p* < 0.01). Correlation analysis is a feasible and convenient method to detect endophytic fungi that potentially promote the accumulation of secondary metabolites in plants [53]. These endophytic fungi have the potential to promote the biosynthesis and accumulation of HupA in plant. Endophytic fungi can serve as biological inducers biological inducers to promote the production of secondary metabolites of host plants by stimulating key genes and synthesizing enzymes in plant biosynthesis pathway [54]. Endophytic fungus *Mucor circinelloides* DF20 promote tanshinone biosynthesis and accumulation in *Salvia miltiorrhiza* root by upregulating the key enzyme genes expression levels of the biosynthesis pathway [18]. Endophytic fungi that promote the biosynthesis and accumulation of HupA in plants and its mechanism need to be further studied.

*Sebacina* was the most dominant community in August. *Sebacina* has been reported to promote plant growth while weakening plant resistance to herbivores [55]. HupA produced by *H. serrata* is an alkaloid that can resist herbivores [56, 57]. In this study, *Sebacina* showed a highly significant negative correlation with the content of HupA in plant (Table S1). It can be speculated that *Sebacina* reduced the content of HupA and damaged the herbivorous resistance of plants, which is the reason for the low content of HupA in August plants.

## Conclusions

Fungal endophytic communities of *H. serrata* varied with season and tissue type, showing high variability in the spatial–temporal distribution patterns. Content of HupA was consistent with the relative abundance of 33 fungal genera across seasons and tissues. It provides a data reference to the promotion of plant HupA biosynthesis.

## Materials and methods

### Plant samples procedure

The natural populations of *H. serrata* materials were collected from Ningqiang County (latitude 32°55′N / longitude 105°55′E, with an altitude of 840 m), Hanzhong City, Shaanxi Province, China and deposited in the Provincial Key Laboratory of Biotechnology, Northwest University (voucher No. NWUHS1708001, NWUHS1711001, NWUHS1802001, NWUHS1805001). The plant material was identified by Prof. Shuonan Wei of Northwest University. According to the local climate, March–May is spring, June–August is summer, September–November is autumn, and December-the following February of the year is winter [46]. *H. serrata* was collected at the end of the four months in August 2017, November 2017, February 2018, and May 2018. We choose to collect mature plants with plant height of 15–20 cm [58]. We randomly selected 5 plants from 30 plants collected each time and merged them into one sample, which was divided into three parts: the root, stem and leaf. Three biological repeats were prepared for each sample, a total of 36 samples.

The whole plants were dug up with the roots and soil, kept the plants moist, placed in sterile self-sealing bags at 4 °C, and the material was processed within 24 h. The surface of the fresh *H. serrata* tissues were washed with water. After removing all spores, the samples were divided into three parts: the root, leaf, and stem. The samples were surface sterilized using the following steps: rinsed with sterilized distilled water for 30 s, soaked in 75% ethanol for 2 min, rinsed 3 times with sterilized distilled water for 1 min each, then sterilized in 0.1% HgCl_2_ for 8 min, rinsed 5 times with sterile water for 2 min each, finally dried with sterile filter paper. The last rinsing water was collected and used as the control. The material was divided into two parts. One part was used for DNA extraction of endophytic fungi, which was quickly frozen with liquid nitrogen and stored at –80 °C, and the other part was used for HupA content detection.

### DNA extraction, PCR amplification and high‑throughput sequencing

Each sample was fully ground in liquid nitrogen, and 1 g was transported to a 1.5 mL centrifuge tube with 700 µL Cetyltrimethylammonium Bromide (CTAB) extract added(β-mercaptoethanol accounts for 0.2% of the extract). Extraction of total DNA from plant materials by CTAB method [59]. The extraction quality of total DNA was verified using electrophoresis on 1.2% agarose gels, and the concentration and purity of DNA were determined by DeNovix DS-11 spectrophotometer (DeNovix Scientific, Delaware, USA).

The intergenic transcribed spacer 1 (ITS1) region of the fungal rRNA genes was amplified with primers ITS1F (5'–CTTGGTCATTTAGAGGAAGTAA–3') [60] and ITS2R (5'–GCTGCGTTCTTCATCGATGC–3') [61]. The PCR mixture (50 µL) contained 200 ng genomic DNA, 2 µL forward and reverse primers (10 µM for each), 5 µL dNTPs (2.5 mM), 10 µL 5 × buffer, and 1 µL TransStart® FastPfu Fly DNA Polymerase (TransGen Biotech, Beijing, China), ddH_2_O to final volume. The PCRs conditions were as follows: 95 °C for 2 min; 30 cycles of 95 °C for 20 s, 58 °C for 20 s, 72 °C for 1 min; a final extension at 72 °C for 5 min. At the same time, set the collected rinsing water as the negative control, and any sample group with bands amplified by the negative control can’t be used for subsequent experiments. The 50 µL amplification products were purified and recovered by 0.8 × volume of VAHTS® DNA Clean Beads (Vazyme, Jiangsu, China), and quantified with a FLx800 Microplate reader (BioTek, Vermont, USA).

The sequencing library was generated using the TruSeq Nano DNA LT library preparation kit (Illumina, California, USA), and the library was purified by 2% agarose gel electrophoresis. Finally, the construction and sequencing of the ITS clone libraries were performed by Personalbio (Shanghai Personal Biotechnology Co., Ltd., China) using an Illumina NovaSeq6000 platform.

### Sequence processing and data analysis

Using DADA2 method, QIIME2 software was used for quality control, denoising, splicing and chimerism removal of sequencing data [62]. The above steps were analyzed for each library. After the denoising of all libraries was completed, the OTUs feature sequences and OTU tables were merged, and singletons OTUs were removed (in all samples, the total number of sequences is only 1 OTU). We used the UNITE database (Release 8.0, https://unite.ut.ee/) [63] to annotate species taxonomy.

In the previous analysis steps, the abundance table of OTU has been generated, and some subsequent analysis steps need to be carried out at the same sequencing depth level. Therefore, the table needs to be transformed. The rarefaction method was adopted, and the flattening depth was set to 95% of the minimum sample sequence size [64, 65].

Python tools are used to visualize the composition and distribution of samples at a specific classification level. We used Krona software (https://github.com/marbl/Krona/wiki) to make an interactive display of the taxonomic composition of the community [66].

In order to comprehensively evaluate the alpha diversity of endophytic fungal communities, Chao [67], observed species, Shannon [68], Simpson [69], faith's PD [70], Pielou's evenness [71] and Good’s coverage [72] indices were evaluated using QIIME2 analysis software. Subgroup samples were plotted as box plots using the Python tool alpha diversity index to visualize the differences in alpha diversity between sample groups. Kruskal Wallis rank sum test and Dunn' test were used to verify the significance of the difference.

Beta diversity analysis was used to compare the similarity between sample communities. Jaccard distance were calculated using QIIME2 analysis software, and then NMDS analysis was done on these distance matrices using Python [73], and the results were plotted as two-dimensional scatter plots. It is generally believed that when the stress value of the NMDS result is less than 0.2, the NMDS analysis result is more reliable [73]. Adonis was used to calculate the significance (p) between the sample groups. Differences were considered significant at *p* < 0.05 and highly significant at *p* ≤ 0.01.

### Extraction and content detection of HupA in plants

The extraction method of HupA from plants has been improved on the basis of traditional methods [74].

The plant materials were dried at 40 °C for 24 h, then ground into powder using liquid nitrogen and dried again. The material was weighed, mixed with 0.5% HCl at a ratio of 1:20 (g/mL), sonicated (500 W for 15 min), then left to stand for 12–14 h. The supernatant was obtained by centrifugation and filtration, and the above extraction steps were repeated twice (without standing), and the supernatants of the three extractions were combined. The supernatant was adjusted to pH 9.0 with NH_4_OH, allowed to stand for 2–3 h, extracted three times using equal volumes of chloroform (2 min of standing and 15 min of sonication each time), and the chloroform of the three times was combined. The chloroform layers were evaporated to dryness with a rotary evaporator under low pressure. The residue was dissolved in 2 mL chromatographic methanol, purified through 0.22 μm Organic microporous membrane filtration and stored in injection vial for standby.

The detection of HupA was performed by high performance liquid chromatography (HPLC) on a Shimadzu LC-20AT (Shimadzu, Tokyo, Japan) high performance liquid chromatograph with a C18 column (250 mm × 4.6 mm, 5 μm; Thermo Scientific, Massachusetts, USA). The mobile phase was ammonium acetate (0.08 mol/L)–methanol–acetonitrile (60:30:10, v/v/v), the flow rate was 0.8 mL/min, the injection volume was 10 μL, and the detection wavelength was 308 nm.

## Supplementary Information

**Additional file 1: Fig. S1.** Krona diagram of all species classification. **Fig. S2.** Heat map of relative abundance of 13 genera of HupA-producing endophytic fungi (a) in three tissues (R, root; S, stem; L, leaf) a (b) four seasons (M8, August; M11, November; M2, February; M5, May). **Table S1.** The negative correlation of HupA content with endophytic fungi within H. serrata (*p* < 0.05).

## Data Availability

The datasets generated and analysed during the current study are available in the National Centre for Biotechnology Information (NCBI) repository [Accession number: PRJNA865097].
