# Supplementary material for: The temporal and spatial endophytic fungal community of Huperzia serrata: diversity and relevance to huperzine A production by the host
Source: BMC Microbiol. 2022 Nov 24;22:281. doi: 10.1186/s12866-022-02702-y (PMC9686072; doi:10.1186/s12866-022-02702-y)
Supplement: Supplementary file 1 — Additional file 1: Fig. S1. Krona diagram of all species classification. Fig. S2. Heat map of relative abundance of 13 genera of HupA-producing endophytic fungi (a) in three tissues (R, root; S, stem; L, leaf) a (b) four seasons (M8, August; M11, November; M2, February; M5, May). Table S1. The negative correlation of HupA content with endophytic fungi within H. serrata (p < 0.05). [file 12866_2022_2702_MOESM1_ESM.docx]

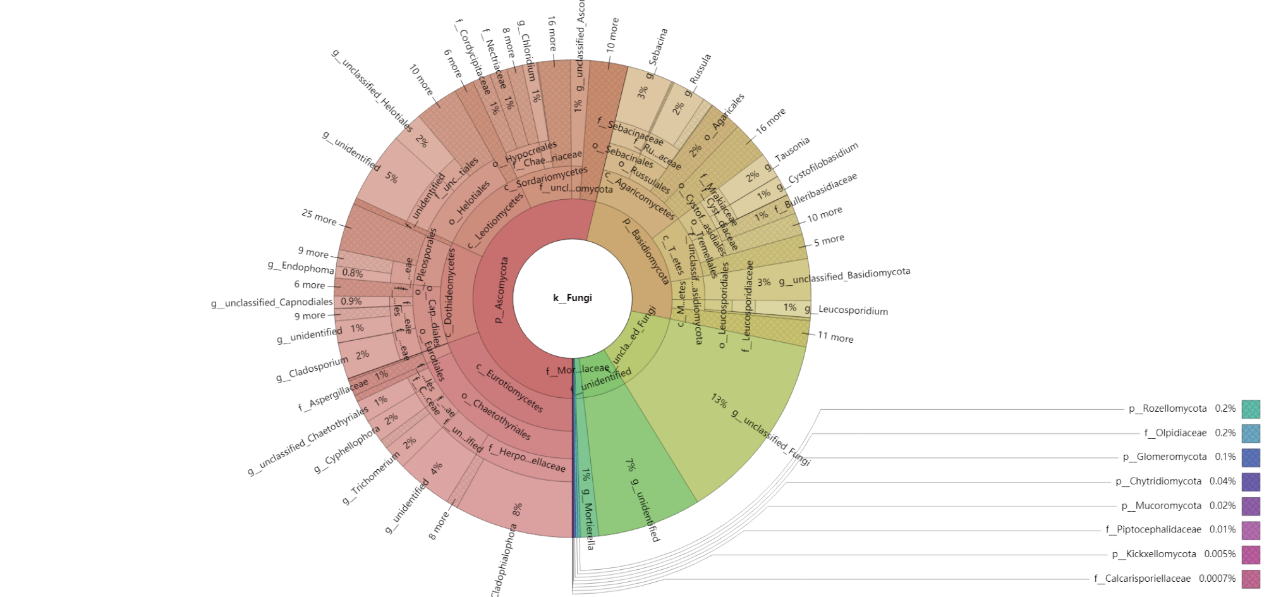


**Fig. S1** Krona diagram of all species classification. The circle of species classification represents the five classification levels of phylum, class, order, family and genus from inside to outside. The size of the fan reflects the relative abundance of different taxons.


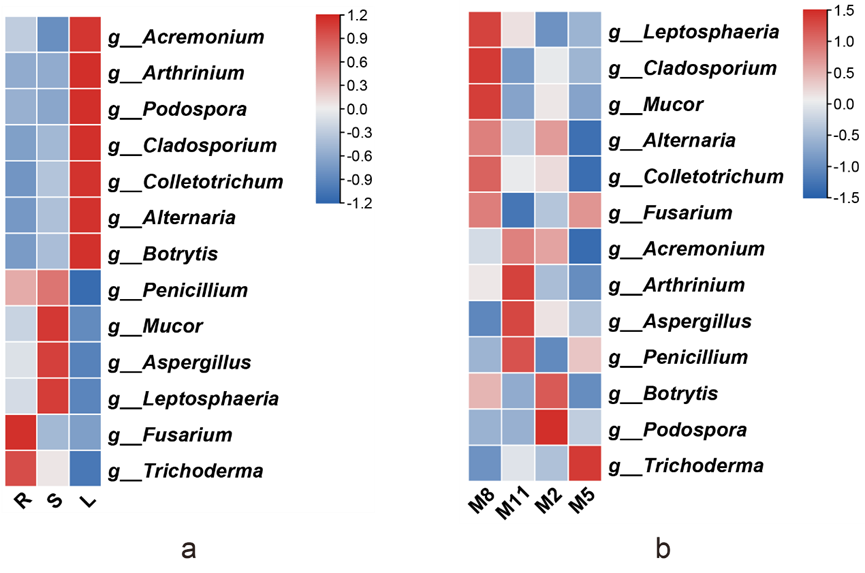


**Fig. S2** Heat map of relative abundance of 13 genera of HupA-producing endophytic fungi (**a**) in three tissues (R, root; S, stem; L, leaf) and (**b**) four seasons (M8, August; M11, November; M2, February; M5, May).

**Table S1** The negative correlation of HupA content with endophytic fungi within *H. serrata* (p<0.05).

| **Genus** | **Spearman’s correlation coefficient** | **Significance（p）** |
| --- | --- | --- |
| *Cadophora* | -0.648342 | 0.000019 |
| *Exophiala* | -0.539611 | 0.000683 |
| *Gorgomyces* | -0.496616 | 0.002064 |
| *Ilyonectria* | -0.485125 | 0.002710 |
| *Sebacina* | -0.482745 | 0.002864 |
| *Saitozyma* | -0.445974 | 0.006408 |
| *Russula* | -0.436634 | 0.007757 |
| *Trichocladium* | -0.421975 | 0.010363 |
| *Lactifluus* | -0.39944 | 0.015801 |
| *Metarhizium* | -0.371738 | 0.025589 |
| *Stemphylium* | -0.36578 | 0.028244 |
| *Leohumicola* | -0.364161 | 0.029003 |
| *Tremella* | -0.360394 | 0.030835 |
| *Pichia* | -0.349479 | 0.036685 |
| *Papiliotrema* | -0.344445 | 0.039671 |
